# Building an Accessible, Low-Cost Cortical Mastoidectomy Trainer: A Technical Report

**DOI:** 10.7759/cureus.97431

**Published:** 2025-11-21

**Authors:** Tanner J Jefferies, Mark K Lavigne

**Affiliations:** 1 Surgery, Campbell University School of Osteopathic Medicine, Lillington, USA; 2 Otolaryngology-Head and Neck Surgery, Scotland Memorial Hospital, Laurinburg, USA

**Keywords:** cortical mastoidectomy, ent training, low-cost model, medical education, otologic simulation, surgical education, temporal bone

## Abstract

Simulation-based training plays a vital role in otolaryngology education, allowing learners to safely and effectively develop fundamental microsurgical skills. However, limited access to cadaveric dissection and high-fidelity temporal bone models due to cost and availability continues to present significant barriers to training in essential otologic techniques such as cortical mastoidectomy. This technical report outlines the design and construction of an accessible, low-cost mastoidectomy trainer intended to support early surgical education. The model is assembled from easily obtainable materials, including a wooden craft ring, a Styrofoam disk, and colored pipe cleaners representing critical anatomical structures such as the tegmen plate and sigmoid sinus. The total assembly cost is approximately $27 USD per unit, with a setup time of less than 20 minutes and no need for specialized tools.

The resulting trainer provides a stable platform for practicing basic drilling concepts, spatial orientation, and the identification of key anatomical boundaries within McEwen’s triangle. Its affordability, ease of construction, and reproducibility make it a practical introductory resource for teaching foundational otologic principles in medical schools, residency programs, and low-resource training environments around the world.

## Introduction

Simulation-based education is an essential component of modern surgical training, enabling learners to develop technical proficiency within a controlled, risk-free environment [1,2]. In otolaryngology, early exposure to simulation has been shown to enhance learner confidence, accelerate skill acquisition, and improve intraoperative safety [3,4]. Among otologic procedures, cortical mastoidectomy remains particularly challenging for early trainees due to its reliance on precise drilling technique, spatial orientation, and recognition of critical anatomical boundaries.

Traditional training modalities, including cadaveric dissection, virtual reality platforms, and 3D-printed temporal bone models, offer valuable educational experiences but are often limited by cost, access to specialized equipment, and institutional resource constraints [5-8]. These barriers disproportionately affect medical students and pre-residency trainees who benefit most from early introductory exposure. Although low-cost surgical simulators have been implemented successfully across several medical disciplines [9,10], there remains a relative scarcity of inexpensive, reproducible, and easily assembled models tailored to otologic education.

Recent work has begun to address this gap. For example, Osorio et al. described a low-cost, easy-to-assemble phonomicrosurgery trainer designed to increase accessibility in early surgical education [11]. This underscores the growing interest in developing affordable, practical models within otolaryngology. However, few existing simulators focus specifically on cortical mastoidectomy, and many available models require specialized equipment, advanced manufacturing techniques, or higher material costs.

This technical report describes the development of a simple, low-cost cortical mastoidectomy trainer intended for early learners, including medical students and pre-residency trainees. The model introduces foundational otologic skills such as basic drilling orientation, hand control, spatial awareness, and identification of anatomical limits within McEwen’s triangle. By clearly defining these educational objectives and emphasizing accessibility, this trainer aims to expand early otologic learning opportunities and support the integration of simulation into undergraduate and introductory residency curricula.

## Technical report

**Figure 1:**
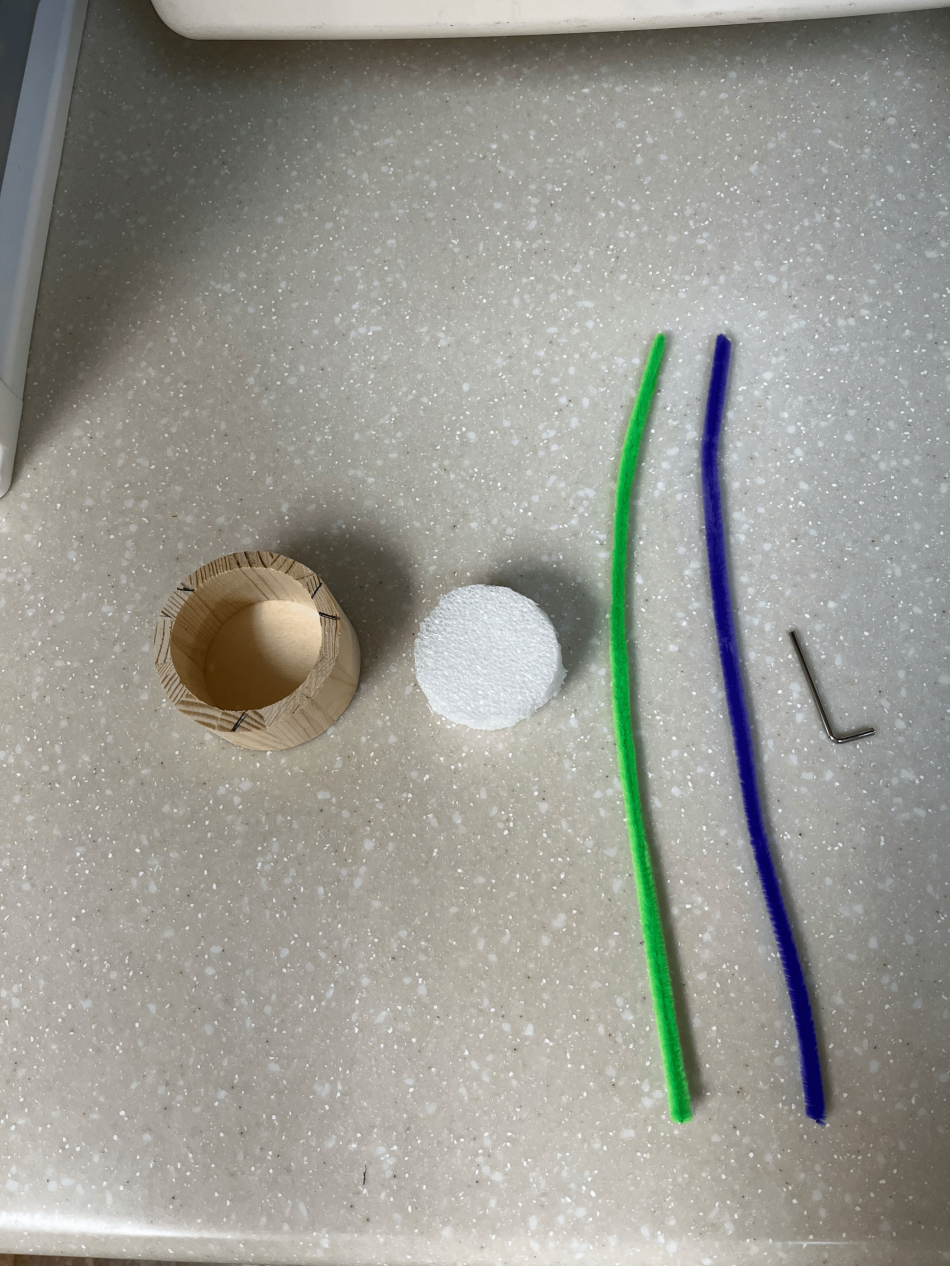
Supplies used for the assembly of the mastoidectomy trainer: wooden craft ring, Styrofoam disk, green and purple pipe cleaners, and small metal rod (Allen wrench)

The cortical mastoidectomy trainer was designed as a simple, low-cost, and reproducible model for introducing early learners to the spatial and technical foundations of otologic surgery. All materials used in its construction are inexpensive, widely available through hardware or craft retailers, and require no specialized tools or fabrication methods (Figure 1). The total material cost is approximately $27 USD per unit (Table 1).

**Table 1:** Cost of supplies for the assembly of the cortical mastoidectomy trainer

Item	Cost
Wooden ring	$1.00
Pipe cleaners (2×)	$0.50
Styrofoam disk	$1.00
Allen wrench	$1.50
Cordless rotary drill (Dremel)	$23.00
Total	$27.00

**Figure 2:**
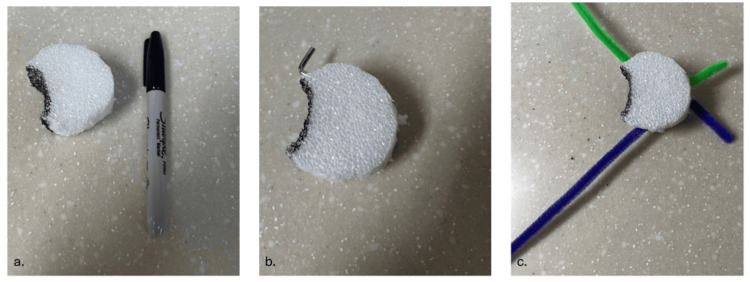
Stepwise assembly of the low-cost cortical mastoidectomy trainer a. Styrofoam disk trimmed and marked with a black permanent marker to create a curved external auditory canal. b. Styrofoam disk with a small metal rod (Allen wrench) inserted to create canals for pipe cleaners. c. Styrofoam disk with embedded pipe cleaners to represent critical structures. The green pipe cleaner simulates the tegmen plate, while the purple pipe cleaner represents the sigmoid sinus.

The outer frame of the model consists of a wooden craft ring that provides structural stability and represents the lateral skull boundary. A circular Styrofoam disk is fitted securely within the ring to simulate the mastoid portion of the temporal bone. The disk is first trimmed and marked with a permanent marker to create a shallow, curved channel representing the external auditory canal (EAC), offering learners an immediate visual landmark during drilling (Figure 2a). 

To incorporate simplified representations of critical anatomical structures, two tunnels are created in the Styrofoam using a small metal rod (Allen wrench) (Figure 2b). Colored pipe cleaners are inserted through these tunnels: a green pipe cleaner represents the tegmen plate, while a purple pipe cleaner represents the sigmoid sinus. Both structures are embedded flush with the surface to provide immediate visual feedback when the learner’s drilling trajectory encroaches upon these boundaries (Figure 2c).

**Figure 3:**
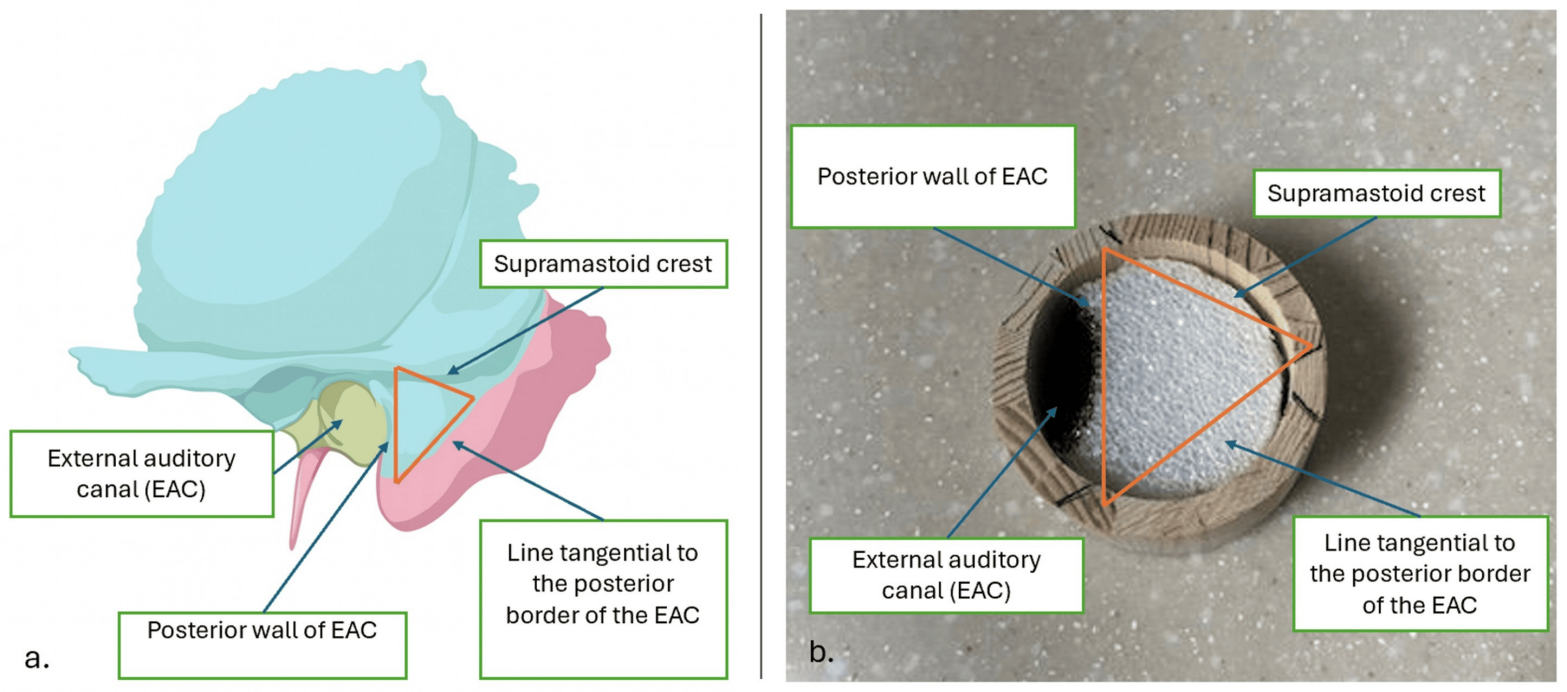
Anatomical and model representation of McEwen’s triangle a. Anatomical illustration demonstrating the key boundaries of McEwen’s triangle, including the supramastoid crest, posterior wall of the EAC, and a line tangential to the posterior border of the EAC. b. Fully assembled low-cost cortical mastoidectomy trainer depicting the same anatomical landmarks represented on the physical model for spatial orientation and educational comparison. EAC: external auditory canal Base anatomical illustration adapted from AO Surgery Reference (CC BY-NC-SA). Original labels removed using AI-assisted editing.

Once assembled, the model provides a stable platform suitable for tabletop use with a handheld rotary drill. The physical properties of Styrofoam offer tactile resistance similar to cancellous bone, allowing demonstration of essential concepts such as burr control, drilling angles, and depth awareness. The model’s configuration also facilitates instruction on identifying the anatomical limits of McEwen’s triangle, helping learners understand spatial relationships within the mastoid cavity (Figure 3). Assembly time is approximately 15-20 minutes, and the Styrofoam insert can be easily replaced to allow repeated practice.

**Figure 4:**
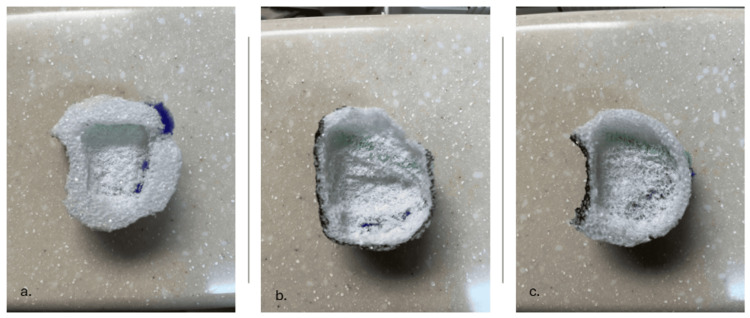
Progressive improvement in drilling technique using the low-cost cortical mastoidectomy model a. First trial showing incomplete cavity formation with short, digging strokes and irregular boundaries. b. Second trial demonstrating improved drilling technique, but with minor dehiscence of the simulated sigmoid sinus. c. Third trial illustrating proper use of long, sweeping strokes, resulting in a broad, well-defined cavity that preserves critical structures, including the posterior wall of the external auditory canal.

The trainer can also serve as a visual and tactile tool for demonstrating progressive improvement in drilling technique. Figure 4 illustrates three sequential trial outcomes performed on the model. The initial attempt shows a shallow, irregular cavity formed with short, digging strokes. The second attempt demonstrates improved technique but includes minor exposure of the simulated sigmoid sinus. The third trial shows the development of consistent, sweeping drilling strokes, producing a smooth, well-defined cavity that preserves key boundaries and simulated neurovascular structures.

Given its affordability, rapid assembly, and reproducibility, this trainer is well-suited for use in undergraduate medical education, residency skills workshops, and resource-limited training environments. It provides an accessible platform for introducing early learners to basic otologic anatomy, spatial orientation, and the fundamental principles of safe drilling technique.

## Discussion

Simulation-based education remains a cornerstone of contemporary surgical training, providing learners with the opportunity to develop technical skills in a controlled, low-risk environment before entering the operating room [1,2]. Within otolaryngology, procedures such as cortical mastoidectomy require precise spatial awareness, controlled drilling technique, and an understanding of critical anatomical boundaries [3,4]. However, access to cadaveric dissection, virtual reality systems, and commercially produced temporal bone models remains limited by financial, logistical, and institutional constraints [5-8]. These barriers highlight the need for accessible, reproducible simulation tools that can support early learner development.

The low-cost cortical mastoidectomy trainer presented in this report offers a practical and accessible contribution to this educational gap. Constructed from readily available and inexpensive materials, the model enables learners to visualize and practice spatial relationships within McEwen’s triangle while receiving immediate visual feedback from embedded representations of the tegmen plate and sigmoid sinus. As demonstrated by the sequential trial outcomes in Figure 4, the trainer can illustrate basic principles of drilling technique, such as the importance of long, sweeping strokes and preservation of anatomical boundaries.

Low-cost simulation tools have been successfully employed across various surgical disciplines to teach foundational psychomotor and cognitive skills [9,10]. Within otolaryngology, interest in accessible, easy-to-assemble trainers has grown, exemplified by Osorio et al.’s low-cost phonomicrosurgery model designed to support early microsurgical skill acquisition [11]. Such work reinforces the importance of affordable educational tools that expand access to simulation. Compared with existing low-cost models, including 3D-printed, resin-based, and hybrid simulators, this mastoidectomy trainer occupies a unique niche by prioritizing ultra-low cost, rapid assembly, and extreme reproducibility. While higher-fidelity models offer more detailed anatomical realism, they often require specialized manufacturing equipment, longer production times, and significantly greater expense.

This trainer is specifically designed for early-stage learners, including medical students and pre-residency trainees, who benefit from simplified platforms emphasizing basic orientation, drilling mechanics, and early spatial recognition. It serves as an introductory tool within a graduated curriculum, complementing, not replacing, cadaveric dissection or high-fidelity simulation later in training.

Although this report focuses on the technical construction and conceptual educational value of the model, no quantitative performance data, learner feedback, or validity measures were collected as part of this initial work. As such, the findings should be interpreted as descriptive and preliminary. Future validation could incorporate structured assessments such as objective performance checklists, timed drilling tasks, or error-tracking metrics to quantify skill acquisition. Pilot usability studies, for example, surveying medical students on realism and utility, would also provide valuable data to support broader curricular integration.

Limitations

This trainer does not replicate the full anatomical detail, variability, or tactile resistance of cadaveric temporal bone. Its simplified design limits its use to introductory instruction and is not appropriate for advanced surgical preparation. The anatomical boundaries represented in the model are conceptual and should be reinforced through cadaveric or high-fidelity simulation before clinical application. Because of its simplified anatomy, the model should be used only for foundational skills, with the understanding that additional training modalities are required to achieve operative competency. Educational validity has not yet been demonstrated and awaits formal evaluation.

Future directions

Future studies will incorporate structured learner assessment, usability surveys, and objective performance metrics to evaluate the model’s effectiveness in teaching early otologic skills.

## Conclusions

The low-cost cortical mastoidectomy trainer described in this report provides a simple, reproducible platform for introducing early learners to the spatial relationships, drilling mechanics, and conceptual anatomical boundaries involved in otologic surgery. Constructed from inexpensive and readily available materials, the model offers an accessible means for medical students and pre-residency trainees to develop basic hand control, orientation skills, and familiarity with McEwen’s triangle in an introductory, low-stakes environment.

As a low-fidelity simulator, this trainer is not intended to replicate the anatomical detail, variability, or tactile properties of cadaveric bone, nor can it serve as a substitute for high-fidelity or cadaveric simulation during advanced stages of training. Its educational impact has not yet been formally evaluated, and further studies incorporating user feedback and structured performance metrics are needed to determine its role within a broader otologic training curriculum. Nonetheless, by lowering financial and logistical barriers, this model supports early engagement in otologic education and may serve as a practical first step in a graduated approach to temporal bone simulation.
